# Mapping High‐TDS Groundwater Near Impoundments Using Ground and Waterborne Towed Electromagnetics

**DOI:** 10.1111/gwat.70041

**Published:** 2025-12-30

**Authors:** Piyoosh Jaysaval, Jonathan N. Thomle, Esteban J. Bowles‐Martinez, Rebecca L. Kreuzer, Frederick D. Day‐Lewis

**Affiliations:** ^1^ Pacific Northwest National Laboratory Earth and Environmental Systems Division, Subsurface Science Group 902 Battelle Boulevard Richland WA 99352

## Abstract

Long‐term monitoring at landfills and impoundments containing coal combustion products (CCPs) or other industrial wastes is essential for detecting possible leachate releases to groundwater and mapping contamination plumes. This study evaluates a novel, non‐invasive geophysical approach—towed time‐domain electromagnetic (TEM) surveys—for non‐invasive and rapid assessment of groundwater quality near landfills and impoundments that have the potential to release plumes with higher total dissolved solids (TDS) than groundwater. CCPs are one such example where releases can have relatively high sulfate, sodium, and/or calcium concentrations resulting in high TDS and, therefore, high electrical conductivity. This makes electromagnetic (EM) methods suitable for their detection and monitoring. Recent advancements in TEM technology enable efficient subsurface imaging over extensive areas using antennas towed by vehicles on land or boats on water bodies. TEM surveys provide valuable information about overburden thickness, geological structures, lithology, and pore‐fluid TDS. We conducted integrated ground‐based and waterborne TEM surveys at a CCP complex adjacent to a river in the eastern United States. Despite challenging site conditions, including railroad tracks, high‐voltage power lines, and power‐generation infrastructure, high‐quality TEM data were collected. Over 20 line‐km of data were acquired and inverted using laterally constrained two‐dimensional (2D) and spatially constrained three‐dimensional (3D) inversions. Results successfully delineated geological boundaries and identified conductive anomalies consistent with elevated TDS indicative of potential leachate plumes. Geophysical interpretations agreed well with water‐quality data from nearby monitoring wells. This work highlights the effectiveness of integrated ground‐based and floating TEM surveys for high‐resolution characterization around CCP impoundments.

## Introduction

Long‐term monitoring at industrial waste sites is critical for the timely detection and delineation of potential contaminated leachate releases into groundwater (Petrovic and Fiket 2022). Coal combustion product (CCP) landfills and impoundments are one such waste site example. Leachate from CCPs typically contains high concentrations of total dissolved solids (TDS), along with inorganic trace contaminants such as boron, lithium, molybdenum, and sometimes arsenic and selenium (EPRI 2021). These elevated TDS levels result in high fluid electrical conductivity relative to native groundwater, making leachate plumes amenable to detection by electrical and electromagnetic (EM) geophysical methods (Singha et al. 2022). Traditional CCP site monitoring relies on groundwater monitoring wells, but these provide only point‐scale data, often missing spatially heterogeneous contaminant plumes. Non‐invasive, rapid, and spatially comprehensive geophysical methods provide a way to map contamination plumes and assess groundwater quality around CCP sites, which enables plume delineation with fewer invasive points and can inform placement of additional monitoring wells as needed. Additionally, there is growing interest in characterizing CCP sites not only for environmental monitoring but also as secondary sources of critical minerals, including rare earth elements (Taggart et al. 2016).

Geophysical EM methods are well established for cost‐effective mapping of subsurface conductivity over extensive areas (Nabighian 1991). EM surveys leverage EM induction to perform non‐invasive measurements of subsurface electrical conductivity, which contrasts with direct‐current electrical methods (e.g., electrical resistivity tomography) that rely on ohmic conduction and thus require physical electrode contact with the ground. EM instruments are broadly categorized into frequency‐domain EM (FEM; e.g., Boaga 2017; Brosten et al. 2011; Jaysaval et al. 2021; Sheets and Hendrickx 1995) and time‐domain EM (TEM; e.g., Stewart and Gay 1986; Nabighian and Macnae 1991; Christiansen et al. 2006; Williams et al. 2021), both of which have been extensively applied in environmental and hydrogeological investigations. Of these, TEM methods, which induce transient EM fields and measure their decay, have demonstrated particular sensitivity to conductive anomalies indicative of contaminated leachate movement (Baawain et al. 2018; Morita et al. 2020; Lu et al. 2025).

Recent technological advancements in TEM instrumentation, particularly the development of mobile, towed systems, have substantially improved survey efficiency and spatial resolution. Ground‐based towed TEM (e.g., the tTEM instrument developed by Auken et al. 2019) systems utilize antennas mounted on sleds or carts pulled by vehicles (e.g., all‐terrain vehicles), enabling continuous and rapid data acquisition over large terrestrial areas (Auken et al. 2019). A complementary waterborne variant, for example, the FloaTEM instrument, extends this capability to aquatic environments by deploying antennas on floating platforms towed by boats (Lane et al. 2020). Both systems are capable of imaging subsurface conductivity structures to depths exceeding 70 m, depending on local conditions such as subsurface conductivity and noise levels (Auken et al. 2019). These towed TEM surveys facilitate integrated imaging across land‐water interfaces, bridging traditional monitoring gaps between terrestrial and aquatic environments and providing comprehensive spatial continuity.

The objective of this study is to evaluate the effectiveness of integrated towed TEM surveys, combining both tTEM and FloaTEM, for characterizing high TDS leachate plumes and underlying geological structures at a CCP site adjacent to a river in the eastern United States. This approach leverages the combined strengths of ground‐based and waterborne TEM surveys, producing subsurface conductivity maps across the land–river boundary. Building upon our preliminary research (EPRI 2023), this study extends prior efforts by incorporating additional analysis and synthetic modeling to support robust interpretation of geophysical results.

## Field Site

**Figure 1:**
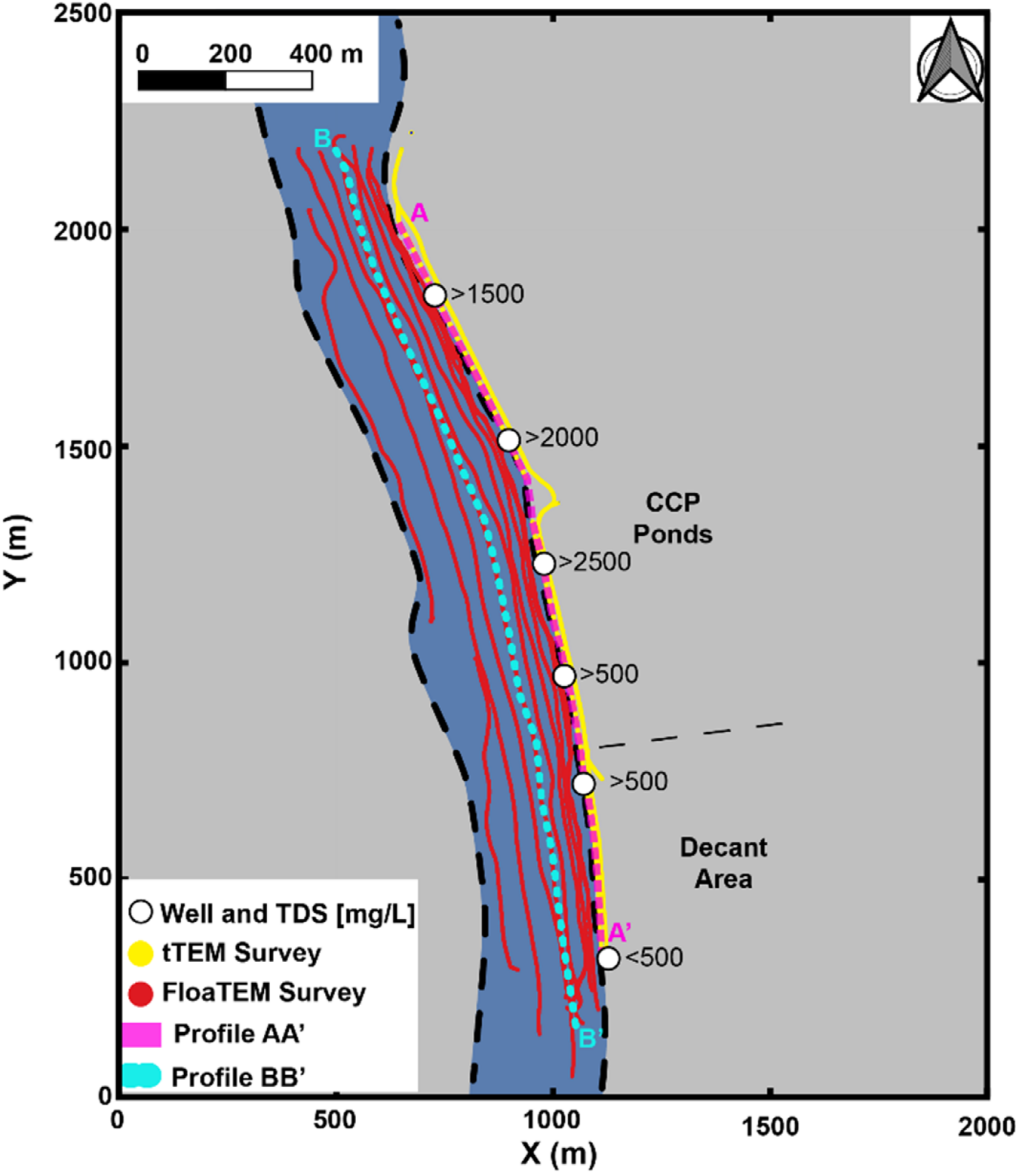
Map of the demonstration site showing the TEM survey lines and well locations. Blue‐filled circles indicate the ground‐based tTEM survey lines conducted along the eastern riverbank, while red‐filled circles represent the waterborne FloaTEM survey lines conducted over the river. Note: markers are closely spaced and appear as continuous lines due to high sampling density. White‐filled circles indicate monitoring well locations with TDS concentrations (mg/L) annotated. Magenta and cyan lines indicate the locations of profiles AA′ and BB′, respectively, considered in later figures. The CCP pond area and decant zone are marked, and the river is shaded in blue.

The field site is underlain by folded and faulted sedimentary rocks composed of alternating layers of limestone, dolostone, shale, sandstone, and siltstone. Soil thickness varies across the site, ranging from approximately 10 to 150 ft. A layer of alluvial sediment of variable thickness mantles much of the site, with deposits between approximately 2 and 30 ft thick, consisting of clay, coarse sand, and occasional boulders. The site is divided by a major thrust fault into northern and southern regions with different hydrogeological characteristics. Groundwater flow is generally toward the southwest, and groundwater discharges to a river that bounds the site to the south and west (Figure 1). Adjacent to the river lies a surface impoundment complex that stores CCPs generated by a nearby coal‐fired power plant. The stored CCPs include fly ash, bottom ash, and flue‐gas desulfurization gypsum. Although detailed site references and geological maps cannot be provided due to sponsor confidentiality, this description reflects the generalized regional setting that guided survey design and interpretation.

The site‐specific objectives of the TEM surveys were: (1) to map subsurface electrical conductivity downgradient of the CCP impoundments and within the riverbed bounding the site to detect possible releases of leachate, and (2) to identify the location of the thrust fault and/or associated geological boundaries, major hydrogeological features, and critical components of the site's conceptual model. An integrated interpretation of ground‐based and waterborne TEM data provides a continuous and coherent understanding of the subsurface across the aquifer–river boundary.

## Methodology

Towed TEM systems enable rapid, high‐resolution, and non‐invasive acquisition of geophysical data across large areas. This section describes the instrumentation used for ground‐based and waterborne TEM surveys, as well as the data processing and inversion workflows employed to generate resistivity models of the subsurface.

### Towed TEM Systems

**Figure 2:**
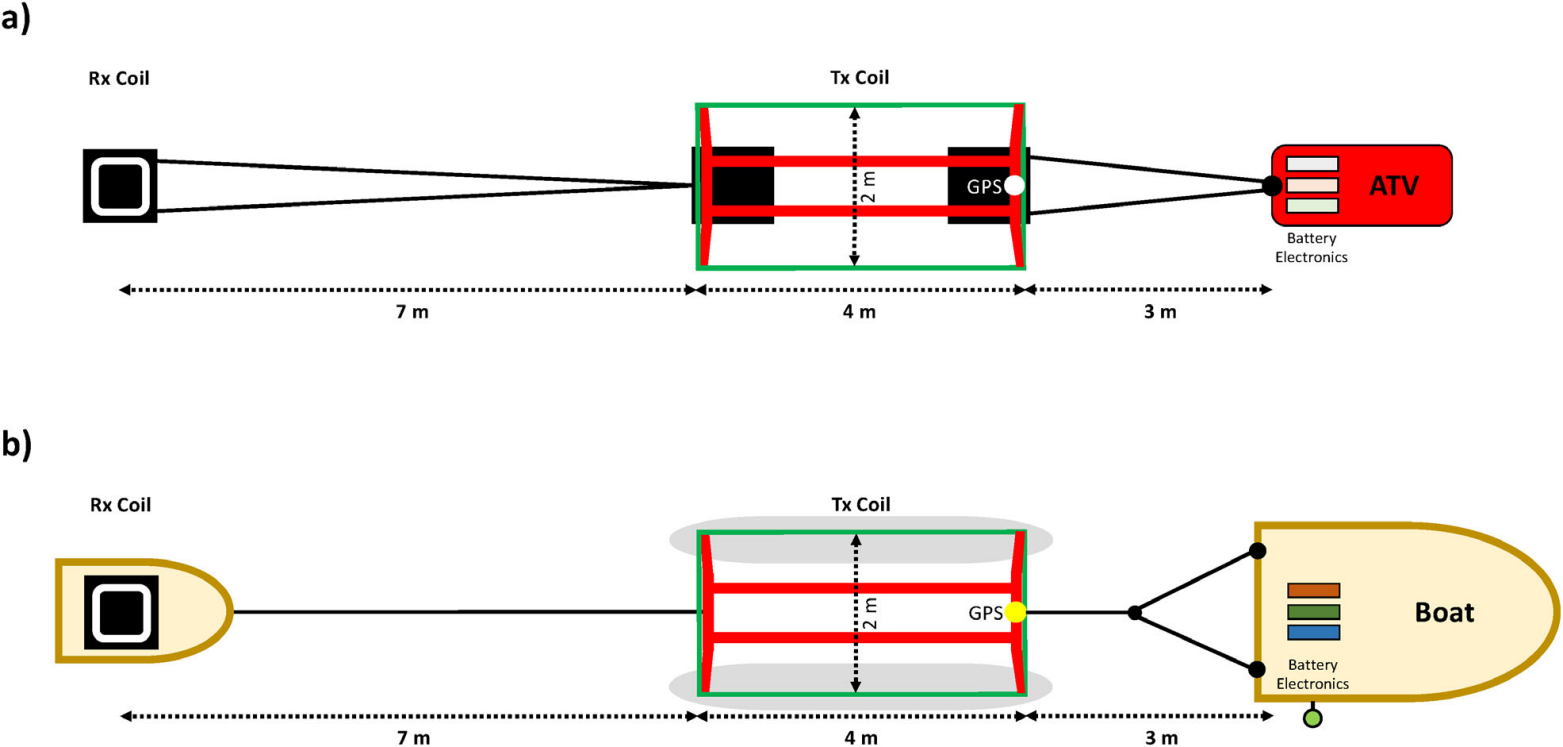
Schematic diagram of the tTEM and FloaTEM systems: (a) tTEM system towed by an ATV on land, and (b) FloaTEM system towed by a boat on water. Tx and Rx coils represent the transmitter and receiver, respectively. Diagrams modified from Auken et al. (2019).

The tTEM and FloaTEM systems (Figure 2) are designed for mapping subsurface electrical conductivity in terrestrial and aquatic environments, respectively (Auken et al. 2019; Lane et al. 2020). The tTEM system is towed behind an all‐terrain vehicle (ATV), while the FloaTEM system is towed by a boat. Both systems offer a lateral resolution of approximately 10 m and a depth of investigation (DOI) typically ranging from the top 2‐3 m to about 50‐70 m, depending on the electrical conductivity of the underlying earth materials or water. In general, DOI decreases in environments with higher background conductivity.

The transmitter (Tx) consists of a 2‐ by 4‐m^2^ single‐turn coil, and the receiver (Rx) consists of a 0.56‐ by 0.56‐m^2^ multi‐turn coil with an effective area of 5 m^2^ (Figure 2). The Tx and Rx are configured as horizontal loops separated by 9 m center‐to‐center in an “out‐of‐loop” configuration (Figure 2). The same Tx and Rx instrumentation is used in both tTEM and FloaTEM systems. In tTEM mode, the Tx and Rx coils are mounted on sleds and towed by an ATV or other similar vehicle, which also houses the battery and electronics (Figure 2a). In FloaTEM mode, the Tx coil is mounted on two paddle boards tethered to a boat, while the Rx coil is placed on an inflatable raft trailing behind the Tx. The boat carries the battery, system electronics, and an echo sounder used to record water depth (Figure 2b). Navigation and data collection are monitored and controlled by the operator using Aarhus Navigator software, which provides a real‐time display of the survey path, system status, Tx current, and positioning. The geographic position of each measurement is recorded using a GPS mounted on the transmitter frame (Figure 2).

In TEM data acquisition, a time‐varying current is passed through the Tx coil, which induces a primary magnetic field in the subsurface. The transmitter current is abruptly turned off, leading to a change in the primary magnetic field, which induces subsequent eddy currents within the electrically conductive bodies of the subsurface. The subsurface eddy currents thereafter induce a secondary magnetic field. The time‐decaying response of the secondary magnetic is recorded at the Rx coil. Because the Rx coil is a horizontal loop, it measures the time derivative of the vertical component of the secondary magnetic field (i.e., db/dt in $VA^{-1}m^{-2}$). The time decay is sensitive to the distribution of the subsurface conductivity, with the early‐time signal sensing the shallower subsurface, and the later time signal sensing deeper.

The system is a dual‐moment system using approximately 3 A and 30 A currents, respectively, for the low‐ (24 Am^2^) and high‐moment (240 Am^2^) measurements. The low moment captures the early‐time signals, and the high moment records the late‐time signals. Each acquisition cycle records 15 time gates for the low‐moment and 30 time gates for the high‐moment recordings. The instrument electronics are thermally stabilized at 45 ±2°C to maintain consistent transmitter current and signal quality.

Typical speeds for both ATV and boat range from 15 to 20 km/h, although lower speeds may be necessary in rough terrain or water conditions. The tTEM/FloaTEM systems collect hundreds of db/dt measurements per second, which are subsequently stacked to produce a single sounding representing an average over a predefined spatial window, typically ~10 m, along the survey line. Depending on field conditions and operating speed, tTEM and FloaTEM surveys can typically cover between 0.5 and 2 km^2^ per day.

### Data Processing and Inversion

Geophysical inversion is used to transform raw TEM data into 2D or 3D images of subsurface electrical conductivity. In this study, we use the Aarhus Workbench software for data analysis. Prior to inversion, the data underwent both automatic and manual preprocessing steps to remove various types of noise, including capacitive coupling from nearby metallic infrastructure, power line interference, and random signal noise. Processed data were then spatially averaged over predefined spatial windows, typically about 10 m, to improve the signal‐to‐noise ratio.

The AarhusInv inversion code (Auken et al. 2015) is then applied to the processed data to obtain cross sections (2D) and volumes (3D) of subsurface resistivity (the reciprocal of conductivity). The inversion process involves solving an optimization problem to estimate a resistivity model that minimizes: (1) the misfit between observed and simulated data in a least‐squares sense at each sounding, and (2) unrealistic spatial variability in the model. The latter is addressed through regularization to mitigate non‐uniqueness in the inversion result. We used laterally constrained inversion (LCI) and spatially constrained inversion (SCI) schemes for 2D and 3D models, respectively, assuming a 1D layered earth model at each sounding (Auken and Christiansen 2004; Viezzoli et al. 2009). The strength of lateral or spatial continuity in the inversion is controlled by user‐defined regularization parameters, which determine the degree of smoothness or sharpness enforced between adjacent 1D models. These parameters are selected based on data quality and geological expectations, balancing spatial coherence with fidelity to observed data. Under these regularization approaches, we seek a resistivity model that matches the data while maintaining consistency between adjacent 1D locations, laterally (2D) or spatially (3D).

Inversions were performed using 25–30 layers with their thicknesses increasing logarithmically with depth. The quality of fit between observed and simulated data is evaluated using the weighted root mean‐squared data residual r (Auken et al. 2019):

(1) $$r=\sqrt{\frac{1}{N}\sum_{i=1}^{N}\frac{{\left(\log d_{\mathrm{obs},i}-\log d_{\mathrm{fwd},i}\right)}^{2}}{e_{i}^{2}}},$$

where $d_{\mathrm{obs}}$ is the observed db/dt data, $d_{\mathrm{fwd}}$ is the simulated data for the estimated resistivity model, e is the standard error in the observed data, and N is the number of data points. A residual r<1 implies that simulated data are fitted within the estimated error bar on the observed data. Values of r≪1 suggest overfitting (i.e., fitting of noise), in which case the inversion would likely show spurious spatial structure. Value of r≫1 would imply underfitting to the data, in which case the inversion would show little spatial structure. Auken et al. (2019) provides additional description of the tTEM/FloaTEM system and data analysis.

The inversion procedure produces 2D and 3D images of electrical resistivity, which are subsequently interpreted in terms of geological structures and variations in pore‐fluid resistivity, which vary directly with TDS. In porous media, lower bulk resistivity is indicative of higher porosity, clay content, and/or fluid electrical conductivity (and thus higher TDS) in the pore space. On the other hand, higher bulk resistivity is indicative of lower porosity, coarser sediments or bedrock, or lower fluid electrical conductivity (and thus lower TDS).

The relationship between bulk resistivity and fluid conductivity in porous media is commonly described by Archie's Law (Archie 1942):

(2) $$\rho_{b}=\rho_{f}\theta^{-m}=\theta^{-m}/\sigma_{f},$$

where $\rho_{b}$ is the bulk resistivity [Ωm]; $\rho_{f}$ is the resistivity of the pore fluid [Ωm]; $\sigma_{f}$ is the conductivity of the pore fluid, that is, the reciprocal of fluid resistivity [S/m]; θ is porosity [−]; and *m* is the cementation exponent [−], which describes the connectivity of the pore space and is generally between 1.5 and 2, with larger values corresponding to less connected, more tortuous, pore space.

It is important to note that Archie's Law assumes non‐conductive earth materials (e.g., sand) and requires modification in the presence of conductive materials (e.g., clay). It should also be noted that direct conversion of inverted resistivity tomograms to fluid resistivity using Archie's Law can produce erroneous results, as tomograms are commonly smoother and show less variability than reality (Day‐Lewis et al. 2005), as discussed in Section 6.

CCP leachate plumes usually have elevated TDS compared to native groundwater and thus are expected to manifest as low‐resistivity features in inversion results (e.g., Harkness et al. 2016). As context for interpretation, we consider the expected bulk resistivity range for a high‐TDS CCP leachate plume. For loosely consolidated sediments, we consider a porosity of θ=0.3 and a cementation exponent of m=1.8 for Archie's Law. The relation between TDS and fluid conductivity is commonly assumed to be linear for fresh‐to‐brackish pore waters, $\mathrm{TDS}=k\;\sigma_{f}$, where the constant of proportionality, k, is ~0.6 (Walton 1989), TDS is in mg/l and $\sigma_{f}$ is in mS/cm. Based on these assumptions, a TDS of 1800 mg/L corresponds to a fluid conductivity of 3000 mS/cm, and this produces a bulk resistivity of 30 Ωm. It is important to note that this example calculation is based on assumptions with respect to Archie's Law parameters; furthermore, the resolution of the inversion results complicates the direct calculation of TDS from bulk resistivity obtained by geophysical imaging (e.g., Day‐Lewis et al. 2005, 2007).

## Field Surveys

**Figure 3:**
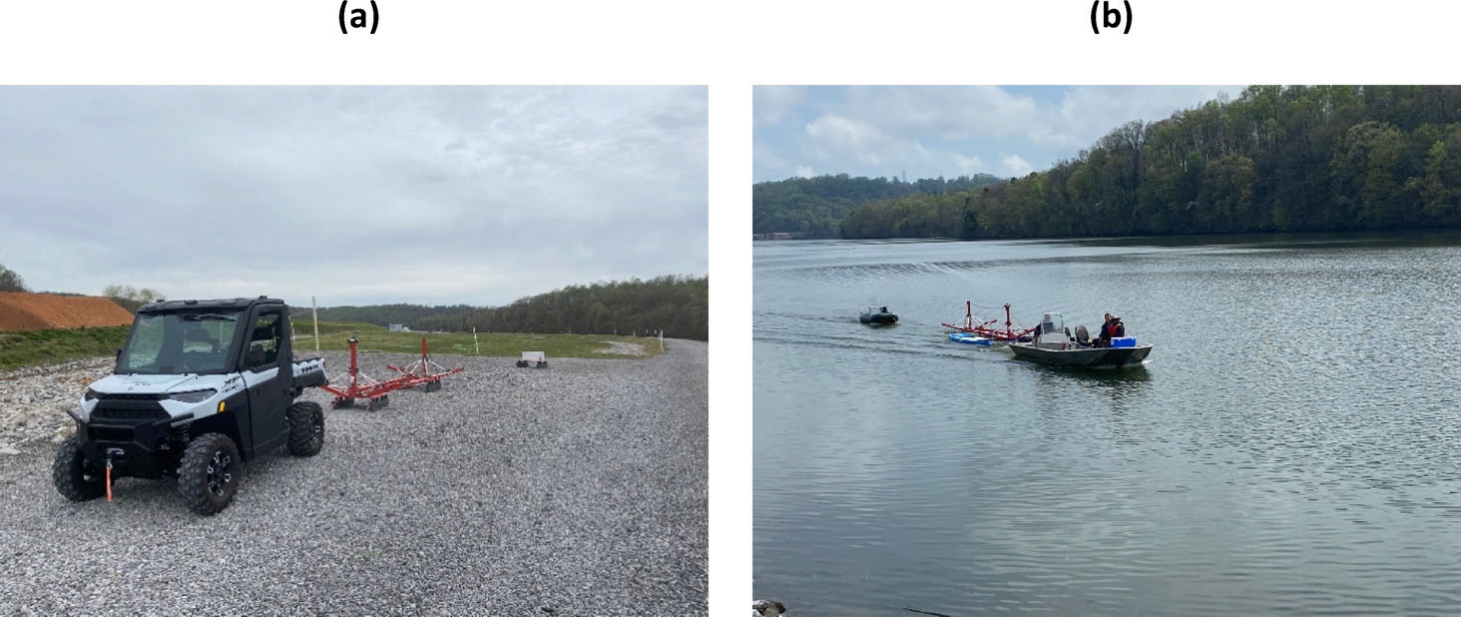
Field photographs showing data acquisition configuration for (a) tTEM and (b) FloaTEM surveys, on land and water, respectively. In (a), Tx and Rx coils are mounted on sleds and towed behind an ATV. In (b), the Tx coil is mounted on paddleboards, and the Rx coil is placed on an inflatable raft, both towed by a boat during waterborne surveys.

In April 2022, ground‐based and waterborne TEM surveys were conducted along north–south oriented transects at the CCP impoundment complex. Figure 1 shows the survey layout, with blue‐filled circles representing the tTEM survey lines collected along the eastern riverbank and red‐filled circles representing the FloaTEM survey lines collected over the river. The tTEM system was towed behind an ATV (Figure 3a), while the FloaTEM system was pulled by a boat (Figure 3b). In total, about 6 line‐km of tTEM data and 16 line‐km of FloaTEM data were acquired, with measurements recorded at intervals of 2 to 3 m. River depths ranging from 2 to 11 m were recorded using an echo sounder attached to the boat during FloaTEM surveys.

The field campaign spanned 3 d. Day 1 was dedicated to system setup and ground‐based tTEM data collection, while Days 2 and 3 focused on FloaTEM data acquisition and system teardown at the end of the third day. Data collection was temporarily halted for several hours on the second day due to thunderstorms in the area.

The test site, located near an active power plant, presented a challenging environment for TEM surveys due to potential EM interference from metallic infrastructure, boreholes, buried utilities, railway tracks, and overhead high‐voltage power lines. Some tTEM data were affected by these noise sources, while waterborne FloaTEM data were relatively unaffected except near the northern extent of the survey area where power lines were present. Noisy or artifact‐laden data were identified and removed during preprocessing. Following data culling, remaining data were stacked using a ~10‐m moving window, as described previously, to suppress random noise and improve overall signal quality. Despite environmental challenges, the overall quality of the TEM dataset was high and suitable for inversion and interpretation. The LCI and SCI regularization were applied to the spatially averaged (~10 m) data, as described in the data processing section.

## Inversion Results

The inversions were performed using 30‐layer models, with the layer thicknesses increasing logarithmically with depth. For the tTEM dataset, the first layer was set to a thickness of 1 m, while for the FloaTEM dataset, the first layer was assigned a thickness equal to the measured water depth from the echo sounder. The final layer thickness for both tTEM and FloaTEM inversions was set to 120 m. Although river water conductivity was measured during the FloaTEM survey, these values were not used to constrain the inversion. Small errors in applied constraints can lead to large inversion artifacts (Day‐Lewis et al. 2007); therefore, the resistivity of the water layer was estimated by the inversion algorithm rather than explicitly fixed.

A homogeneous half‐space model with a resistivity of 45 Ωm was used as the starting model for both tTEM and FloaTEM inversions. LCI and SCI regularization were applied in tTEM and FloaTEM inversions, respectively.

**Figure 4:**
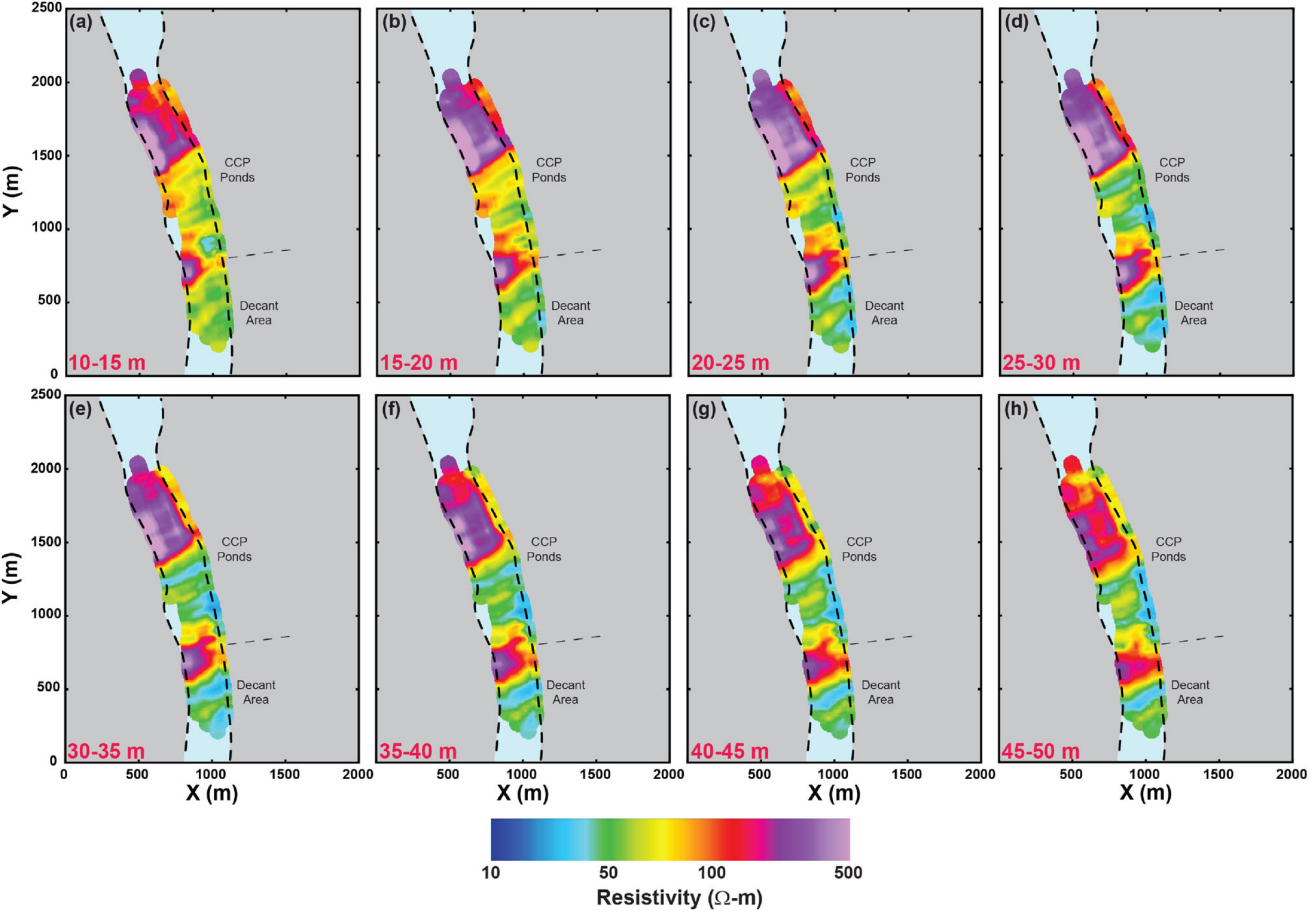
Inverted resistivity images averaged over different depths: (a) 10–15 m, (b) 15–20 m, (c) 20–25 m, (d) 25–30 m, (e) 30–35 m, (f) 35–40 m, (g) 40–45 m, and (h) 45–50 m. These images are obtained from the FloaTEM dataset.

**Figure 5:**
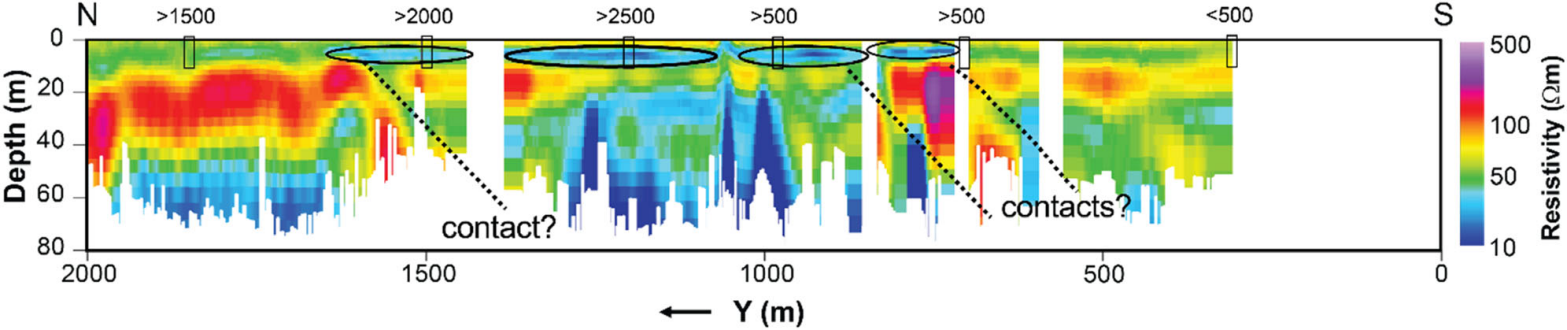
Vertical cross section of the inverted resistivity image along profile $A-A^{\prime }$ (Figure 1) obtained by inverting the tTEM dataset. The ellipses mark the location of a shallow low‐resistivity region consistent with high‐TDS pore water. Black dotted lines indicate inferred geological contacts.

**Figure 6:**
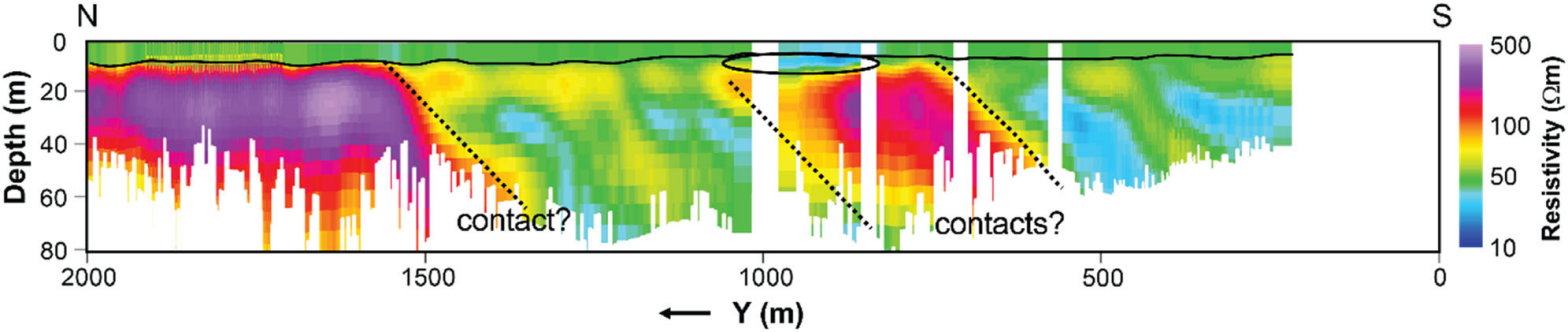
Vertical cross section of the inverted resistivity image along profile $B-B^{\prime }$ (Figure 1) obtained by inverting the FloaTEM data. The solid black line indicates the river bottom. The ellipse marks the location of a shallow low‐resistivity region consistent with high‐TDS pore water. Black dotted lines indicate inferred geological contacts.

The inversion results produced good agreement with the observed TEM data. Most weighted data residuals (Equation 1) were less than 1, indicating that the modeled data closely matched the observed signals within their respective error bounds. Figure 4 shows horizontal slices of the average resistivity beneath the river derived from the FloaTEM dataset. Vertical cross sections of resistivity along the transects A‐A′ and B‐B′ are shown in Figures 5 and 6. In all figures, cool colors (e.g., blue tones) correspond to low resistivity values, whereas the warm colors (e.g., violets and purples) represent high resistivity values.

## Synthetic Modeling

In this section, we present a suite of synthetic modeling exercises to evaluate the sensitivity of the tTEM and FloaTEM systems to key geological features and high‐TDS leachate plumes. The results of such synthetic modeling provide a conceptual framework for the interpretation of inversion results and help geophysicists and end users develop intuition regarding spatial resolution and potential inversion artifacts. We use forward modeling codes to produce synthetic data for different scenarios, add reasonable noise to the data thus generated, and then use inverse modeling to analyze the noisy data. We consider synthetic models that mimic the resistivity structures shown in Profile B–B′ (Figure 6). Using data acquisition parameters consistent with our field data collection, synthetic TEM responses are calculated for the synthetic models. To simulate field conditions, 5% random noise is added to the synthetic responses. The noisy data are then inverted using a homogeneous 45 Ωm half‐space as the initial model. It is not expected that the recovered model will be an exact match to the original synthetic model because of the regularized inversion, the diffusive nature of subsurface EM fields, and noise added to the data.

**Figure 7:**
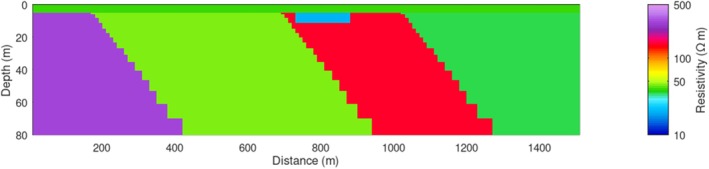
Synthetic resistivity model representing the conceptual geological framework along Profile B–B′ (Figure 6). The model includes background resistivity structures, a 4.9‐m water column, and a 6.2‐m thick low‐resistivity anomaly (20 Ωm) inserted in the streambed to simulate a high‐TDS leachate plume.

**Figure 8:**
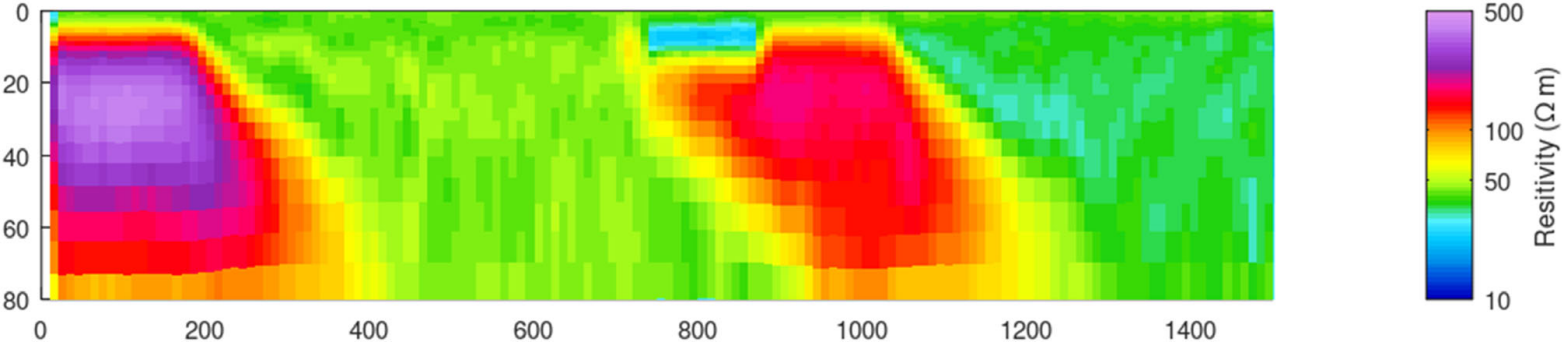
Inverted resistivity cross section obtained from synthetic data with 5% added random noise, corresponding to the model shown in Figure 7. The inversion result replicates key features of the true synthetic model, including the shallow low‐resistivity anomaly (~20 Ωm) and geological boundaries (e.g., dipping faults), demonstrating the system's capability to resolve high‐TDS plumes and structural features.

In the synthetic modeling, we consider a water depth of 4.9 m and include (1) a low‐resistivity anomaly of 20 Ωm in the streambed with a thickness of 6.2 m, based on the shallow low‐resistivity feature (representing high‐TDS plume) found in Profile B–B′ (Figure 6), and (2) several dipping geological contacts, based on those interpreted in Profile B–B′ (Figure 6). The synthetic model is shown in Figure 7. The estimated cross section obtained from the inversion of the synthetic data is shown in Figure 8, which was smoothed with a 3 × 3 moving average filter after inversion to reduce speckling. The inversion fit the data with a normalized residual of 1.01.

This synthetic modeling supports our interpretations of dipping geological contacts and a possible high‐TDS plume, as annotated in Figures 5 and 6. Inversion of synthetic TEM data based on synthetic models resolves geological structures and plume features and produces structures similar to those observed in field data. The assumed shallow conductive feature in the synthetic model manifests in the inversion with a similar size, shape, and resistivity to that found in the tomogram based on field data. The geological background formations are also very similar in overall resistivity and in intra‐formation variability to those in the model produced by inverting the true data.

Additional modeling was done to test the sensitivity of this method by adjusting the depth and thickness of the conductive feature and evaluating the inversion's ability to resolve it. The above analysis places the feature at the streambed, and it continues downward for four model layers to reach its 6.2‐m thickness. We modified these parameters by shifting the top of the layer downward by one, two, and three layers, corresponding to depths below the riverbed of 1.2, 3.0, and 5.5 m, respectively. We also adjusted the thickness of the conductive feature, using one, two, and three model layers to define its thickness. Because the thickness of each model layer increases with depth, the exact thickness of each of these anomaly models depends on the depth of the top of the feature; thus, a feature comprising one model layer is between 2.3 and 3.0 m thick, a feature comprising two layers is between 3.7 and 4.8 m thick, and a feature comprising three layers is between 5.3 and 6.7 m thick.

**Figure 9:**
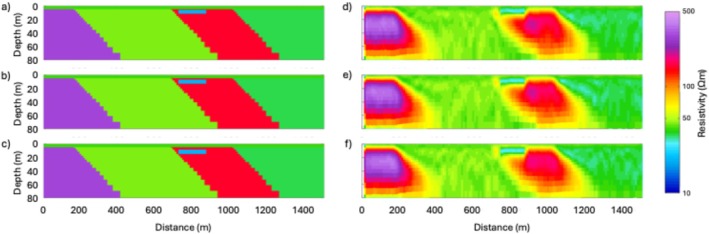
Sensitivity analysis showing synthetic models (left column) and corresponding inversion results (right column) for cases where the low‐resistivity anomaly is three model layers thick and progressively buried deeper by shifting its top boundary downward by 1, 2, and 3 layers, approximately 1.2, 3.0, and 5.5 m below the riverbed, respectively, shown in panels (a), (b), and (c). Corresponding inverted models (d–f) maintain clear visibility of the anomaly, indicating that resolvability is not significantly affected by modest increases in burial depth.

We see that all three of the deeper three‐layer features (Figure 9) are resolved similarly to the one in Figure 8, though a slightly higher resistivity is visible in these compared to the one in Figure 8. Overall, moving the feature deeper has minimal difference in resolvability compared to when the feature is at the streambed.

**Figure 10:**
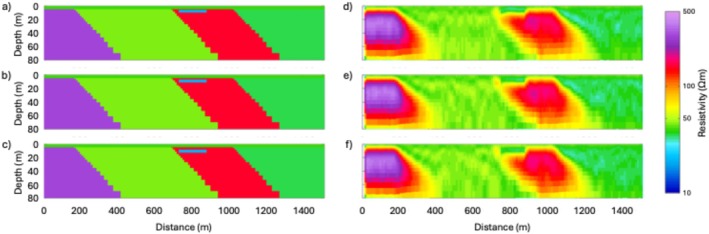
Synthetic and inverted models for scenarios where the low‐resistivity anomaly is reduced to two layers in thickness (~3.7–4.8 m), with the top of the anomaly positioned at three different depths below the riverbed. Synthetic models are shown on the left in panels (a–c), and corresponding inversion results are shown on the right in panels (d–f). Compared to the thicker anomalies in Figure 9, the resistivity contrast is less distinct, indicating reduced sensitivity to thinner features.

**Figure 11:**
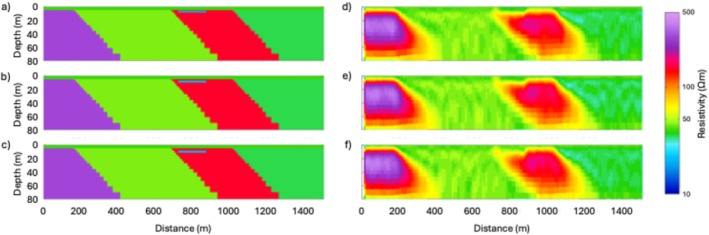
Synthetic (a–c) and the corresponding inverted (d–f) models for cases where the low‐resistivity anomaly is reduced to a single model layer in thickness (~2.3–3.0 m) and positioned at three different burial depths below the riverbed. Compared to Figures 9 and 10, the anomaly becomes less distinct and more difficult to resolve, particularly at greater depths. These results highlight the strong dependence of anomaly detectability on its thickness.

Thinning the feature to two model layers, however, does make a noticeable difference (Figure 10). These thinner features still show a low‐resistivity zone, but the resistivity contrast is not as clear as with the thicker models presented in Figure 9. This effect is even more pronounced when the feature is only one layer thick (Figure 11). In these models, resistivity is still suppressed, but the feature is not as clear and only slightly different from background resistivity. If these features were in a less resistive formation, they might be overwhelmed by noise and unresolvable. What we can see from these three sets of synthetic tests is that the resolvability of a high‐TDS groundwater plume depends more on its thickness than on its depth. We also see that a 6‐m feature produces an anomaly in the inversion results similar to the anomaly resolved by the field results (Figure 6), suggesting that the true low‐resistivity feature is of a similar thickness.

## Discussion

Interpretation of the tTEM and FloaTEM inversion results (Figures 4, 5, 6 through 6) is guided by the synthetic modeling presented in Figures 7, 8, 9, 10, 11 through 11 and performed with consideration for the hydrogeological context and research objectives: detecting and delineating geological features and potential high‐TDS groundwater plumes. It is well understood that geophysical inversion has inherent limitations in spatial resolution, and inversion results often exhibit smoothing and blunting effects (Day‐Lewis et al. 2005). As a result, sharp contrasts associated with lithological boundaries appear more gradational than in reality. The resistivity values estimated by inversion are blunted, overestimating low values and underestimating high values relative to the true distribution of resistivity in the earth.

A particular challenge in this study was the lack of boreholes and water‐quality sampling data directly beneath the river, which limited our ability to ground‐truth the FloaTEM inversion results. Resistivity anomalies in the streambed could result from a variety of sources, including variations in lithology or soil type, sediment texture, pore‐fluid conductivity (e.g., due to TDS), or a combination thereof. For this interpretation, we assume that (1) high‐TDS plumes would appear as low‐resistivity anomalies at shallow depths, and (2) limestone and shale formations would appear, respectively, as high and low‐to‐moderate resistivity features, with contacts between them dipping to the SE, according to regional geological structure. The results displayed in Figures 4, 5, 6 through 6 are interpreted in this framework.

**Figure 12:**
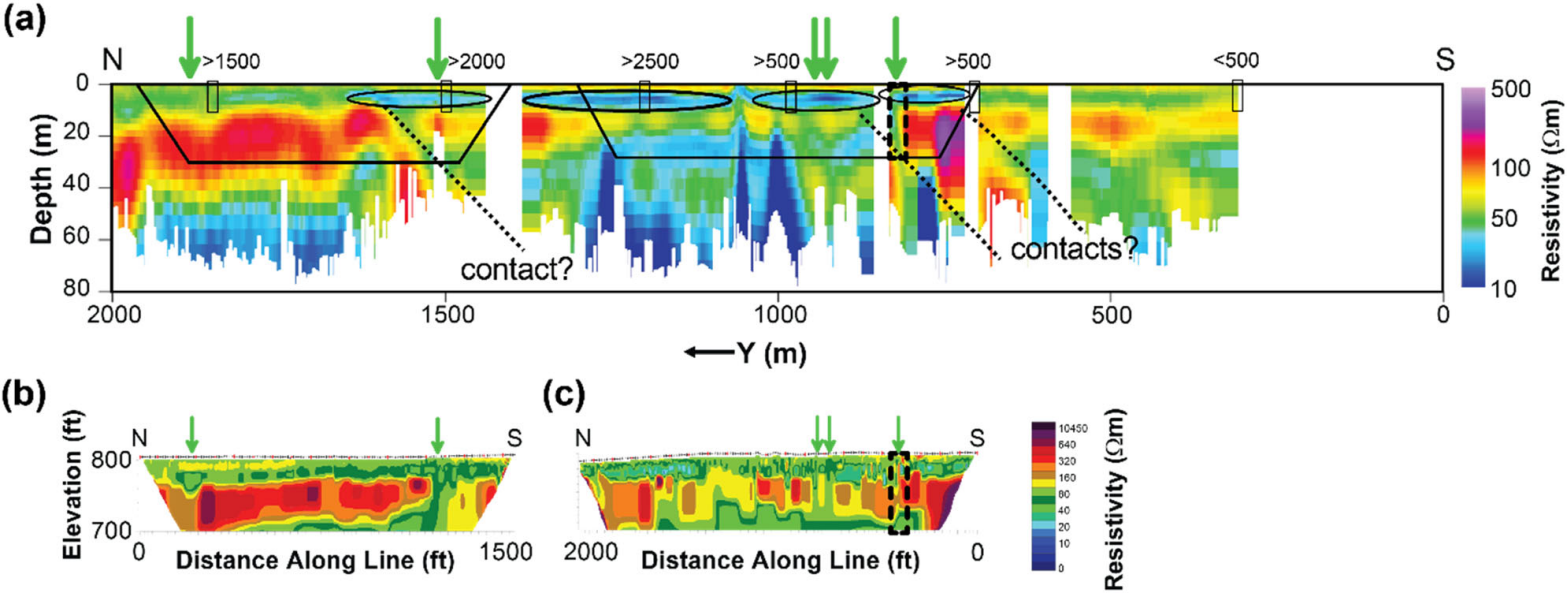
Comparison of tTEM and ERI surveys: (a) tTEM along profile A‐A′, repeated from Figure 5; (b and c) ERI results from previous investigations, positioned below their corresponding locations along the tTEM transect. The ERI results are legacy data with a different resistivity scale; comparisons are qualitative. The approximate locations of ERI sections are delineated by black polygons on the tTEM profile. Resistivity anomalies identified previously from ERI are indicated by green arrows on both ERI and tTEM profiles. A potential fault zone interpreted from the ERI study is indicated by a box with a dashed black border on both ERI and tTEM profiles. Monitoring wells near A‐A′ are projected to the transect and indicated by black rectangles with generalized sampling results for TDS (mg/L). Note differences in units and color scales between the TEM and ERI results.

TEM results are compared with water‐quality sampling (TDS) where observation wells are located and with electrical resistivity imaging (ERI) results from a previous study contracted by the site owner (Figure 12). Compared to the ERI surveys, the TEM survey covered a much larger area and greater DOI. Where the surveys overlapped, the TEM and ERI results show strong consistency, with most ERI low‐resistivity anomalies appearing in TEM results; however, the tTEM survey appears to detect shallow low‐resistivity features better than the nearby ERI transect. The TEM surveys better resolved dipping features, the orientations of faults or contacts, and deeper resistivity variations. We attribute the comparative advantages of TEM to (1) the smaller distance between the transmitter and receiver for the tTEM/FloaTEM (~9 m) compared to the longer length of an ERI electrode array (~100+ m), and (2) the greater DOI afforded by the tTEM/FloaTEM (~70 m) system compared to the ERI survey (~30 m).

### Faults and Geological Boundaries

The dipping interfaces between high‐ and low‐resistivity regions in Figures 5 and 6 are interpreted as geological boundaries that may represent either lithological contacts or structural features such as a major thrust fault mapped in this area. This fault strikes NE–SW and dips to the SE at an angle of approximately 5–20 degrees, consistent with the apparent dips to the SE at ~15–20 degrees interpreted from the resistivity sections. Other boundaries with similar orientation (Figures 5 and 6) may correspond to additional geological contacts, minor shear zones, or folds. We interpret low‐resistivity anomalies that do not extend upward to shallow depths (<20 m) below the river surface (Figure 6) as indicative of geological nature rather than being related to TDS.

### High‐TDS Porewater

A low‐resistivity layer seen in the tTEM results (Figure 5) at depths of ~5 to 10 m and extending from *y* = ~700 m to *y* = 1700 m is interpreted as one or more zones of high‐TDS groundwater; this anomaly is consistent with water‐quality sampling from nearby wells. Data from monitoring wells near the tTEM transect indicate several locations of elevated TDS of 2000 mg/L or higher (Figure 5). With one exception, these low‐resistivity features do not appear to extend into the streambed in FloaTEM results (Figure 6). Although several low‐resistivity features are observed in the streambed near the eastern bank adjacent to CCP impoundments (Figure 4a to 4c), most are deep (>10 m) and extend to the DOI. Additionally, Figure 6 shows similar features having similar orientations. Hence these are interpreted as geological boundaries. The one exceptional anomaly (*x* = ~900 m, *y* = ~800 m, $\rho_{b}=\sim 30\mathrm{\Omega m}$) appears only in the shallowest horizontal slice (Figure 4a) in the FloaTEM inversion results, above a high‐resistivity region (Figure 6); this anomaly does not appear to be geological in nature and is interpreted as high‐TDS pore fluid (~1800 mg/L using Archie's Law and assumptions previously discussed). Furthermore, this feature coincides with two similarly shallow low‐resistivity anomalies in the tTEM results (Figure 5) inland. This low‐resistivity zone is interpreted as indicating high TDS in streambed porewater; this interpretation is consistent with sediment porewater sampling conducted in this area during a previous investigation. At the time of the TEM survey, the site owner was performing corrective action in this area to address the issue.

### Comparison to Electrical Resistivity Imaging (ERI)

ERI was used previously at the study site. Like TEM, ERI images subsurface electrical resistivity structure, but the measurement physics differs. Whereas TEM requires no physical coupling of antennas to the ground and thus is non‐invasive, ERI requires galvanic coupling of electrodes (i.e., metal stakes typically) to the ground. ERI is, therefore, more labor‐intensive, and data acquisition is slower. The DOI for TEM depends on transmitter moment, which is a function of size and power, whereas the DOI for ERI is a function of the distance between electrodes. In our field demonstration, the tTEM and FloaTEM surveys achieved a ~60‐m DOI using a ~9‐m antenna array (Figures 2 and 3). To achieve the same DOI, an ERI survey would require a 300‐m electrode array, which is labor‐intensive to set up and impractical in some settings on land and cumbersome to pull behind a boat in smaller or meandering streams. On land, mobile TEM data acquisition of several line‐km/h is practical in most settings, but for ERI, data acquisition is slower and more labor‐intensive. With ERI, each survey line of electrodes (spanning tens to hundreds of meters) requires an hour or more to set up, several hours for data collection, and an hour or more to break down.

Where both ERI and TEM results are available for comparison, there is general consistency (Figure 12). The ERI results indicated low‐resistivity anomalies in the same areas as tTEM, although not as clearly. Inference of deeper geological structure benefited from the superior DOI for TEM, which more clearly revealed dipping structures. Comparing the TEM and ERI images, TEM provided superior resolution of vertical structure, with ERI smoothing low‐resistivity anomalies across depth.

## Conclusions

The large spatial extent of typical CCP complexes, the physical heterogeneity of deposited materials over that extent, and proximity to water bodies pose challenges to direct groundwater sampling and plume delineation, thus motivating interest in the use of non‐invasive geophysical and remote‐sensing methods. Many CCP sites pose additional challenges to geophysical characterization because of the presence of underground and overhead utilities and EM noise sources associated with powerplant infrastructure and power transmission. In a field demonstration at a CCP complex adjacent to a river, we assessed mobile TEM (i.e., tTEM and FloaTEM) as an approach to support hydrogeological characterization and plume detection. Despite considerable EM noise from nearby infrastructure (e.g., power lines, railroads, infrastructure, and buried pipes), high‐quality data were obtained in both ground‐based and floating surveys over much of the study area. Analysis of the TEM data revealed subsurface resistivity structures interpreted as geological contacts, which likely influence groundwater flow and transport. In addition, shallow low‐resistivity anomalies were identified, consistent with zones of elevated TDS, indicative of potential CCP leachate plumes. Synthetic modeling supported the interpretation of these anomalies and confirmed the system's sensitivity to high‐TDS features. Compared to ERI geophysical surveys performed previously at the study site, TEM provided greater DOI, enhanced resolution of geological structures, and better detection of high‐TDS pore fluids.

## Data Availability

Research data are not shared.
